# Ackerman Unmanned Mobile Vehicle Based on Heterogeneous Sensor in Navigation Control Application

**DOI:** 10.3390/s23094558

**Published:** 2023-05-08

**Authors:** Chi-Huang Shih, Cheng-Jian Lin, Jyun-Yu Jhang

**Affiliations:** 1 Department of Computer Science and Information Engineering, National Chin-Yi University of Technology, Taichung 411, Taiwan; 2 Department of Computer Science and Information Engineering, National Taichung University of Science and Technology, Taichung 404, Taiwan

**Keywords:** heterogeneous sensor, Ackerman unmanned mobile vehicle, deep learning, object detection, navigation control

## Abstract

With the advancement of science and technology, the development and application of unmanned mobile vehicles (UMVs) have emerged as topics of crucial concern in the global industry. The development goals and directions of UMVs vary according to their industrial uses, which include navigation, autonomous driving, and environmental recognition; these uses have become the priority development goals of researchers in various fields. UMVs employ sensors to collect environmental data for environmental analysis and path planning. However, the analysis function of a single sensor is generally affected by natural environmental factors, resulting in poor identification results. Therefore, this study introduces fusion technology that employs heterogeneous sensors in the Ackerman UMV, leveraging the advantages of each sensor to enhance accuracy and stability in environmental detection and identification. This study proposes a fusion technique involving heterogeneous imaging and LiDAR (laser imaging, detection, and ranging) sensors in an Ackerman UMV. A camera is used to obtain real-time images, and YOLOv4-tiny and simple online real-time tracking are then employed to detect the location of objects and conduct object classification and object tracking. LiDAR is simultaneously used to obtain real-time distance information of detected objects. An inertial measurement unit is used to gather odometry information to determine the position of the Ackerman UMV. Static maps are created using simultaneous localization and mapping. When the user commands the Ackerman UMV to move to the target point, the vehicle control center composed of the robot operating system activates the navigation function through the navigation control module. The Ackerman UMV can reach the destination and instantly identify obstacles and pedestrians when in motion.

## 1. Introduction

With the advancement of science and technology, the development and application of unmanned mobile vehicles (UMVs) have emerged as topics of crucial concern in the global industry. The development goals and directions of UMVs vary according to their industrial uses, which include navigation, autonomous driving, and environmental recognition; such uses have become priority development goals for researchers in various fields. UMVs recognize the external environment through sensors, which each have different advantages and disadvantages [1]. Various sensor combinations and weather conditions [2] affect the mobile performance of UMVs. Commonly used sensor types include cameras, radars, and LiDAR (laser imaging, detection, and ranging).

Camera sensors easily identify objects using high-resolution color information. Gradual advancements in image processing technology have contributed to the rapid development of the deep learning field. Researchers have widely employed convolutional neural networks (CNNs) to achieve satisfactory results in various vision applications. Girshick [3] proposed the detection method of region-based-CNN (R-CNN). R-CNN extracts input images and identifies 2000 bounding-box object region candidates; the proposed bounding-box images are then resized and input to the CNN for feature extraction. Then, a support vector machine (SVM) [4] classifier is used to classify and regress bounding-box objects. Although R-CNN boasts high classification accuracy, it runs slowly and requires a long training process. To achieve real-time image detection, researchers have developed one-stage object detection models with an end-to-end design and a fast execution speed, such as the Single Shot MultiBox Detector [5] and You Only Look Once (YOLO) [6]. YOLO uses a CNN to simultaneously predict multiple object frames and calculates the confidence level of each. Numerous scholars have developed a series of network models [7,8,9,10,11] based on YOLO to boost accuracy and optimize the detection ability of small objects [11].

Radar sensors obtain information about the physical environment, such as the relative speed, distance, angle, and direction of motion of high-precision targets. Valtl et al. [12] proposed an artificial neural network navigation algorithm for autonomous driving based on frequency-modulated continuous-wave radar. They employed 60-Hz frequency-modulated continuous-wave radar to obtain environmental information, gathered training data through vehicle manipulation, and used a deep CNN [13] to convert the information; steering and speed were set as the network outputs. Ort et al. [14] developed an autonomous navigation system based on localizing ground penetrating radar (GPR) that can operate in harsh weather conditions. They employed existing GPR technology to interpret combinations of soil, rocks, and tree roots beneath the vehicle.

Although LiDAR and radar operate similarly, LiDAR emits laser light instead of millimeter waves. LiDAR resolution is inferior to that of cameras, but it can obtain clearer object outlines compared with radar. Mihai et al. [15] proposed a machine learning-based pedestrian detection system using 16-line LiDAR. The application of linear interpolation compensates for the low resolution of LiDAR and enables real-time detection of pedestrians. Gao et al. [16] proposed a dynamic clustering algorithm for LiDAR obstacle detection in autonomous driving systems. Generating a region of interest allows the algorithm to filter out unnecessary point cloud data. Clustering is then implemented to identify potential obstacles. Due to the influence of the scanning mechanism, the spatial distribution of the point cloud is not uniform. Gao et al. thus proposed a dynamic clustering algorithm based on the analysis of the spatial distribution of point clouds along different directions to achieve higher detection accuracy. Jianmin et al. [17] proposed a road obstacle detection system for unmanned vehicles based on multilayer LiDAR; this system extracts road edge data sets from LiDAR data and performs cluster analysis. The Dezert–Smarandache theory is employed to construct the environment grid map and detect dynamic obstacles through the collision coefficient in the drivable area. Finally, the extended algorithm completes cluster analysis and information extraction of the dynamic obstacles.

The mainstream research of UMV systems focuses on the combined use of multiple sensors to exploit their complementary advantages and disadvantages. Potdar et al. [18] proposed a method to accomplish localization, obstacle detection, and path planning in an Ackerman steering robot [19,20] using a single camera and LiDAR sensor; LiDAR data are transmitted over a wireless network to a computer using the TCP/IP protocol, and obstacle data from the robot are augmented with potential fields and combined with positional data from overhead cameras to construct cost maps for use in navigational algorithms. Four different search algorithms [21,22,23] were implemented for testing. The results revealed that the hybrid A* navigational algorithm outperformed the three other methods. Weon et al. [24] employed multi-sensor data fusion to develop an autonomous driving method that matches 3-D LiDAR data with 2-D image data. To remove noise in 3-D LiDAR data, the random sample consensus method is used to segment between the ground and objects, and interpolation is employed to match 2-D images with 3-D data.

Navigational algorithms have been widely employed in UMVs. Dijkstra [25] proposed Dijkstra’s algorithm, which identifies the shortest path in a known environment and finds the least cost path between two nodes. This algorithm searches from the starting point to the rest of the map and sets cost points. When the algorithm identifies the next lowest-cost waypoint, the cost of the neighbor is updated. Hart et al. [22] proposed the A* search algorithm, which is similar to Dijkstra’s algorithm; however, it adds a score to the end-point distance and combines the movement cost and end-point distance to identify the next moving point. Rösmann et al. [26] proposed the timed-elastic-band (TEB) algorithm, which treats the path as an elastic rubber band affected by a deforming force; the force includes all the constraints on the robot’s motion and minimizes the trajectory execution time. Fox et al. [27] proposed the dynamic window approach. This strategy samples multiple velocities in the velocity space, simulates the expected trajectories of each set of velocities, and scores each trajectory. Aguilar et al. [28] design a path planning algorithm for a UGV of Ackermann. The developed NHR-RRT algorithm was used for path planning to the best path that is closer to the final goal. Results show that the Ackerman unmanned ground vehicle reaches the final point in a certain time. Wang et al. [29] applied DeepLabV3+ semantic segmentation to realize the classification of road scene images. The automatic controller of the Ackerman unmanned vehicle is established using the cascade PID model. Experiment results show that the proposed method helps the decision system to better judge the current vehicle situation and make appropriate decisions. Carpio et al. [30] present an effective navigation architecture for Ackermann vehicles in orchard environments. The pose regulation controller and navigation controller are implemented in ROS. Experimental results demonstrate reliability even in the presence of sudden dynamic obstacles along the planned route.

Most of the literature emphasizes the design of navigation algorithms and obstacle avoidance methods for Ackerman unmanned vehicles. However, many dynamic obstacles in the real environment are not considered. If obstacles and pedestrians can be effectively identified, collisions between vehicles and pedestrians can be avoided. Furthermore, most of these methods rely on a single sensor (e.g., camera, infrared, LiDAR), which leads to the poor robustness of Ackermann UGV. To improve those shortcomings, this study proposes a fusion technique with heterogeneous imaging and LiDAR sensors in an Ackerman UMV. The major contributions of this study are as follows:

❖ A fusion technique involving heterogeneous imaging and LiDAR sensors is proposed.
❖ YOLOv4-tiny and simple online real-time tracking (SORT) are used to detect the location of objects and perform object classification and tracking to ensure that the objects encountered by vehicles are pedestrians or static obstacles.
❖ LiDAR is employed to obtain real-time distance information of detected objects. Compared with other sensors (infrared, sonar, etc.), LiDAR is less affected by the environment and has high precision.
❖ The vehicle control center (VCC) activates the navigation control module based on heterogeneous sensors in real time. The VCC vehicle can perform obstacle avoidance and navigation functions, allowing the vehicle to reach its destination.
❖ The experimental results indicated an average distance error of 0.03 m and an average error of the entire motion path of 0.357 m when using LiDAR in the Ackerman UMV.

This paper is organized as follows. Section 2 introduces the architecture of the Ackermann UMV system and the navigation control method based on heterogeneous imaging and LiDAR sensors. Section 3 presents the experimental results of the developed Ackermann UMV system. In addition, single-sensor and multi-sensor performance analysis are discussed. Section 4 describes the conclusions of this study and provides recommendations for future research.

## 2. Materials and Methods

**Figure 1:**
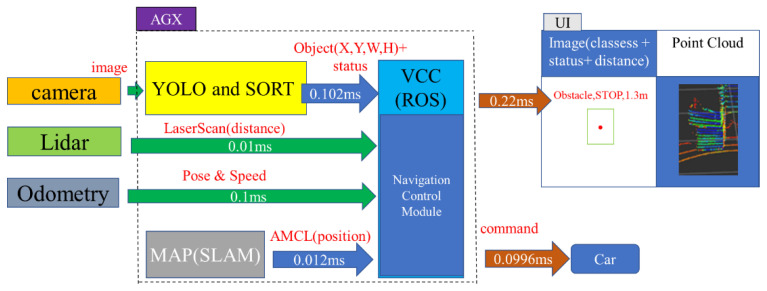
Architecture of proposed Ackerman UMV system.

This study developed an Ackerman UMV system with heterogeneous sensors. The proposed system architecture is presented in Figure 1. As shown in Figure 1, the fusion of heterogeneous data involving camera images, laser imaging, laser distance, vehicle pose, and speed in the Ackerman UMV system has achieved better performance in vehicle control. The proposed Ackerman UMV system works online for real-time control. The latencies of each function are shown in Figure 1. The YOLO and SORT algorithm takes 0.102 ms, the laser scan takes 0.01 ms, odometry takes 0.1 ms, SLAM takes 0.012 ms, navigation control takes 0.0996 ms, point cloud computing takes 0.22 ms, and the total calculation time of the system is 0.5436 ms.

**Figure 2:**
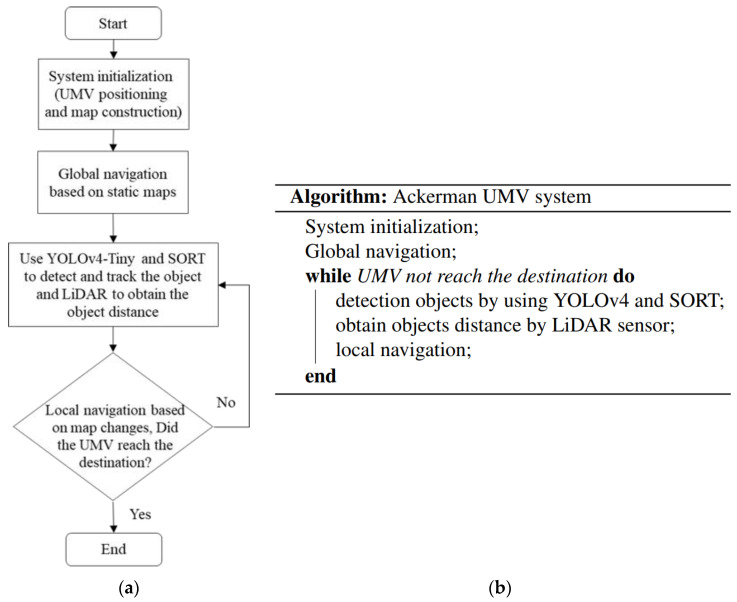
(**a**) The flowchart and (**b**) the pseudocode of the Ackerman UMV system.

During system operation, the camera obtains real-time images; it then employs YOLOv4-tiny to determine the position, classification, and movement of objects. LiDAR simultaneously gathers real-time distance information. To avoid delays caused by excessive image data and point cloud data, this system uses the NVIDIA AGX Jetson Xavier embedded system for calculation. An inertial measurement unit (IMU) is used to collect odometry information to calculate the position of the Ackerman UMV. Static maps are created using simultaneous localization and mapping (SLAM). When the user issues a command to the Ackerman UMV to move to the target point, the VCC composed of the robot operating system (ROS) uses the navigation control module to activate the navigation function and simultaneously identifies obstacles and pedestrians in real time. The system flowchart and pseudocode are presented in Figure 2.

### 2.1. Hardware Architecture of the Ackerman UMV

**Figure 3:**
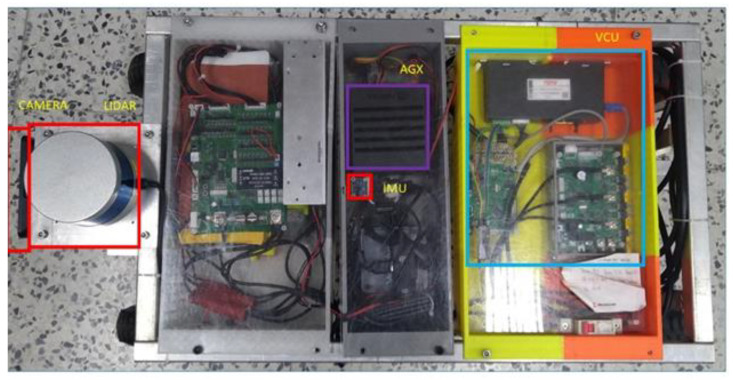
Location diagram of related components in Ackerman UMV.

The Ackerman UMV is composed of sensors, computing devices, controllers, and a vehicle chassis. The vehicle chassis includes the steering system, the power transmission system, and the frame. The VCC is the control terminal, and the AGX embedded system acts as the computing device. The AGX collects sensor information, makes decisions, and issues commands to the VCC to control the power and steering systems. Figure 3 is a location diagram of related components in the Ackerman UMV.

This study employed NVIDIA Jetson AGX Xavier as a computing device; it collects sensor information, makes decisions, and sends commands to the onboard controller to operate the power and steering systems. The operating system is Ubuntu 18.04. ROS provides common functions, management of functional blocks, communication between blocks (publish/subscribe), and device controls. The camera, a Logitech C925e with a 45° horizontal field of view, is installed on the front center of the vehicle. The camera is used to input real-time images to analyze the identification process of obstacles in front of the vehicle, such as pedestrians. The LiDAR sensor, which uses a Velodyne VLP-16 with a 360° horizontal viewing angle, is mounted on the front center of the vehicle above the camera. The sensor is used to construct maps and obtain real-time distance information. The IMU uses a Bosch imu_bno055, which can calculate the acceleration relative to the three axes as an input of the odometry information.

The steering system of the UMV adopts Ackermann steering. The servo motor is connected to and fixed on the Ackermann lock point, converting the rotational motion of the motor into linear motion. The steering rod is used to pull the steering column and change the direction of the tires. The transmission mode is designed to drive the rear wheels. The gears of the gearbox are driven by the motor, and the decelerated power is transmitted to the transmission shaft to directly control the rear wheels.

### 2.2. Ackerman UMV Positioning and Map Construction

SLAM enables the Ackerman UMV to start from an unknown location in an unknown environment, identify its own position through repeatedly observed map features during movement, and construct a map based on its position. Cartographer, a real-time indoor SLAM method developed by Google, supports multi-sensor mapping. This study employed Cartographer to construct a static map for the input of the system navigation module.

### 2.3. Navigation Control

**Figure 4:**
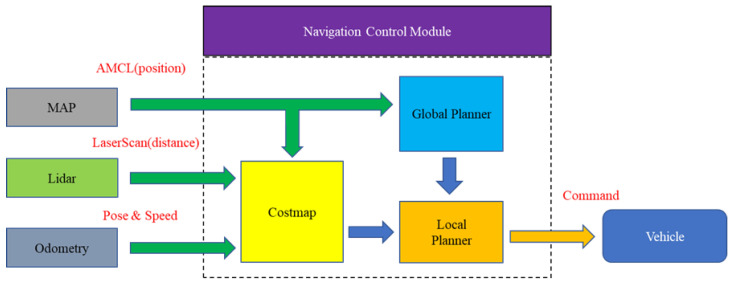
Architecture diagram of system navigation control module.

Navigation control is a crucial system function used to determine the best route and avoid collisions. Figure 4 presents the architecture diagram of the system navigation control module.

First, global navigation is employed to establish a predetermined path, and then local navigation is used to correct and optimize the path through a cost map. The path with the highest score is converted into linear and angular velocities and commands the Ackerman UMV to move. The result of the final navigation is then completed. The cost map is constructed with the static map, dynamic map (including map and obstacles), and odometry information. Cartographer provides simultaneous 2-D and 3-D localization and mapping in real time. This study employed Cartographer to obtain a static map; adaptive Monte Carlo localization is used to locate the Ackerman UMV on the map. LiDAR is employed to obtain angular distances around the Ackerman UMV. The IMU is used to obtain the odometry information, which includes the position of the Ackerman UMV (position and rotation angle [X, Y, θ]) and speed (including linear speed and steering speed). Global navigation uses Dijkstra’s algorithm to identify the shortest path, and local navigation implements the TEB algorithm to optimize the path given to global navigation. Dijkstra’s algorithm starts at the source node and analyzes the graph to find the shortest path between that node and all the other nodes in the graph. If the shortest path between the source node and another node is found, that node is marked as “visited” and added to the path. The process continues until all the nodes in the graph have been added to the path. Then the shortest path can be obtained. Finally, the motion path of the Ackerman UMV is determined.

### 2.4. Object Detection and Tracking Based on YOLOv4-Tiny and SORT

**Figure 5:**
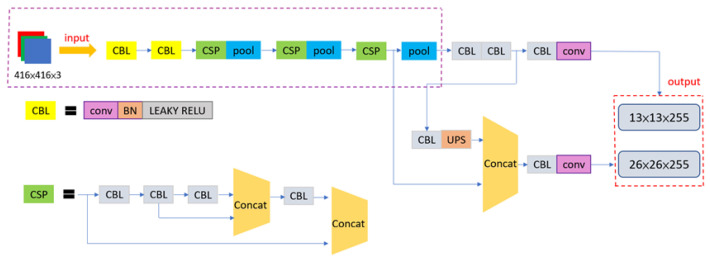
Network architecture diagram of YOLOv4-tiny.

In the Ackerman UMV system, the YOLOv4-tiny network model is used to detect obstacles and pedestrians. YOLOv4-tiny, a lightweight network simplified from YOLOv4, uses the CSPDarknet53-tiny network to extract object features. UpSampling and Concat are used to merge previous features to expand feature information and improve detection performance. In this network, predictions use scale sizes of 13 × 13 and 26 × 26. Figure 5 presents a network architecture diagram of YOLOv4-tiny.

**Figure 6:**
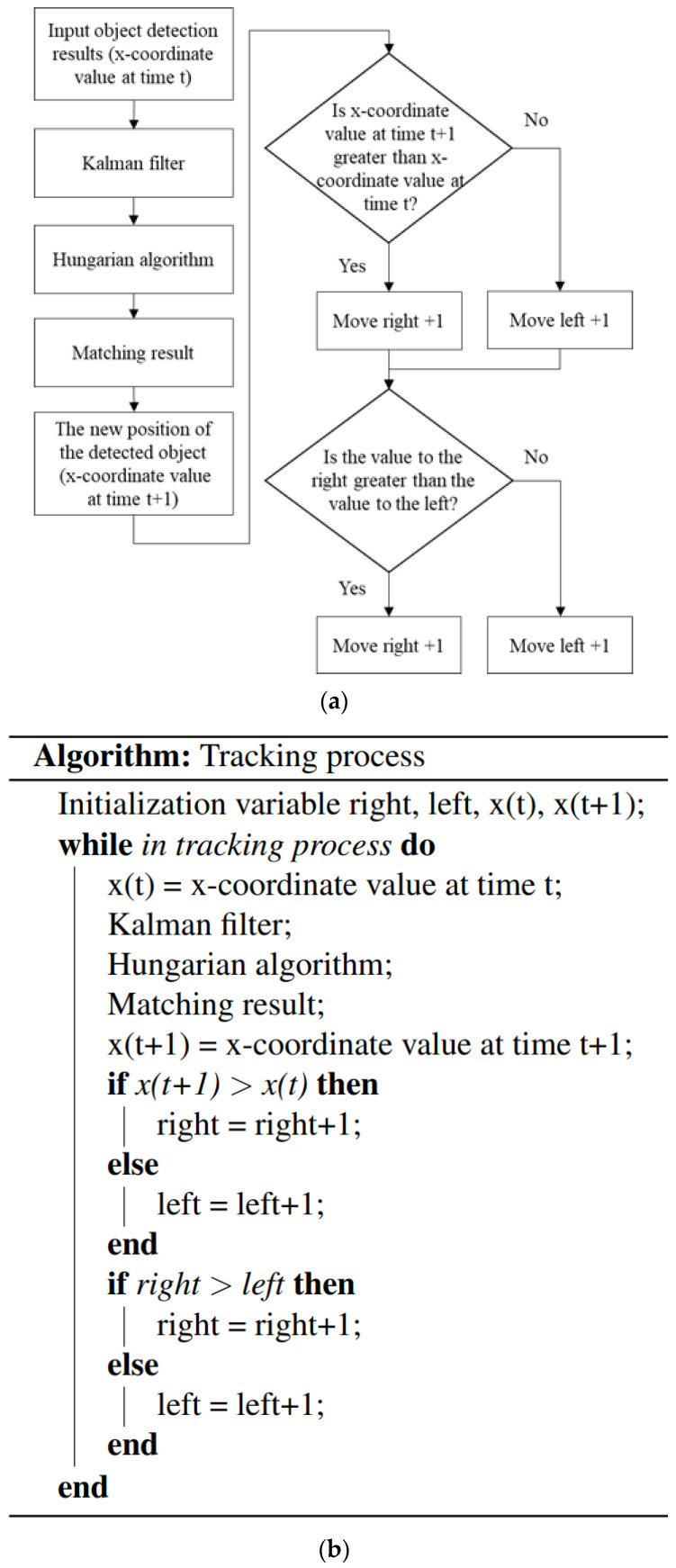
(**a**) The flowchart and (**b**) the pseudocode of the system tracking process.

If the direction of pedestrian travel can be determined, the probability of collision with the Ackerman UMV can be reduced. Therefore, this study implemented correlation matching of detected objects in different image frames to determine whether detected objects were new. SORT [31] was used to track dynamic objects (Figure 6).

### 2.5. Integration of LiDAR and Imaging

**Figure 7:**
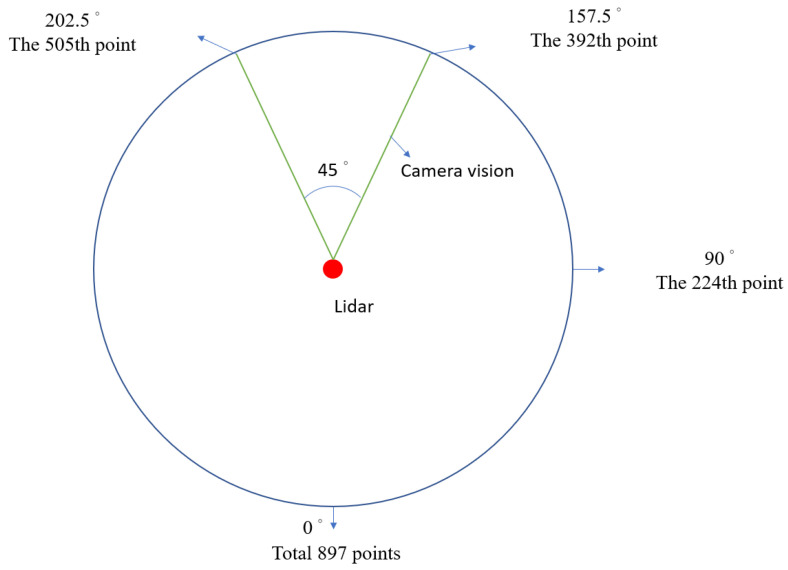
LiDAR sensor and camera visual angle.

**Figure 8:**
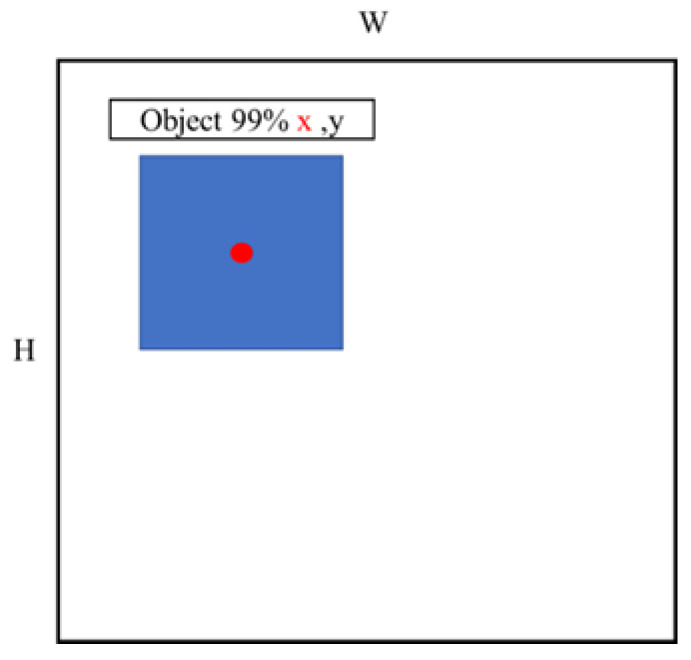
Integration of LiDAR sensor and imaging.

Each laser layer of LiDAR can return 897 distance points and be evenly distributed in 360°. To determine the distance between the obstacle and the vehicle in the image recognition system, the camera view angle and the LiDAR sensor must be matched. Figure 7 presents a schematic of the LiDAR and the camera visual angle. The red ball indicates the LiDAR sensor, and the angle between the two green lines is the visibility range of the camera. The distance interval (DI), which is the range of the camera visual angle and the LiDAR point, is obtained. When YOLOv4-tiny detects an object, it determines the object’s detection frame and center point. The *X*-axis ratio of the center point to the pixel identifies the corresponding angle from the DI. Figure 8 presents a schematic of the integrated LiDAR and imaging technology. The window width (W) is 480 pixels, the window height (H) is 640 pixels, and x and y are the coordinates of the center point.

The corresponding value of the *DI* is the distance between the red point and LiDAR; the distance (*Dis*) formula can be expressed as follows:

(1) $$Dis=DI*\left(\frac{x}{W}\right)$$

**Figure 9:**
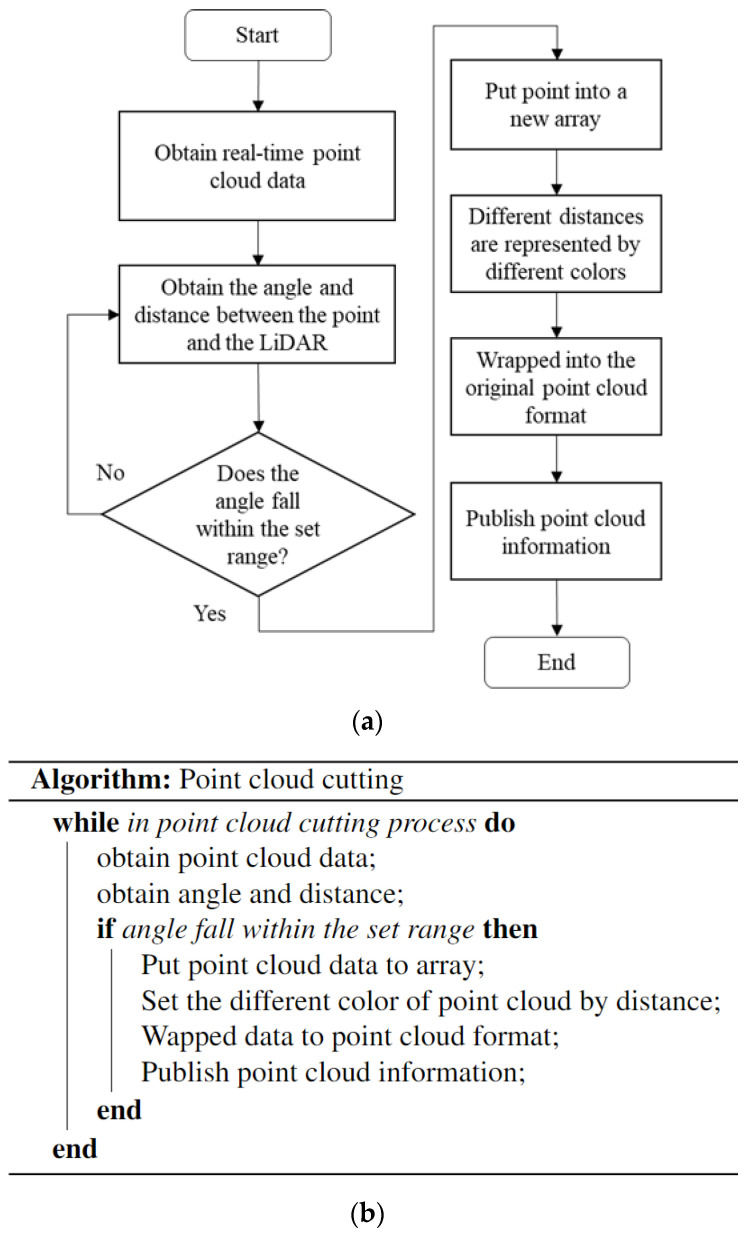
Point cloud cutting process. (**a**) Point cloud cutting flowchart. (**b**) Point cloud cutting pseudocode.

To obtain the point cloud and image comparison, a LiDAR and image integration method is used to cut the point cloud and implement point cloud analysis of the camera visual angle range. Figure 9 presents a flowchart of the point cloud process.

**Figure 10:**
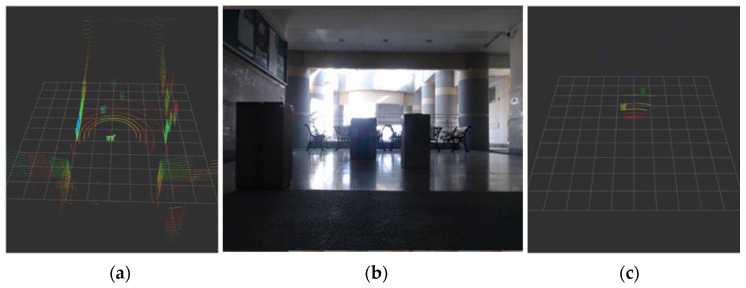
(**a**) Camera visual angle. (**b**) Original point cloud data. (**c**) Cutting result.

When the LiDAR sensor scans a circle, it obtains point cloud data. The data contain information (*x*, *y*, *z*, *I*) about a point, where *x*, *y*, *z* are the coordinates corresponding to each axis, and *I* is the intensity of the laser reflection. In Figure 10, we used a planar method to identify the coordinates that correspond to the LiDAR sensor and the camera. After identifying the *x* and *y* coordinates, the angle (*θ*) and distance (*D*) between the point and the LiDAR sensor are calculated; the formulas are represented as follows:

(2) $$\theta =tan\left|\left(\frac{x}{y}\right)\right|$$

(3) $$D=\sqrt{x^{2}+y^{2}}$$

If the angle falls within the set range, the distance data are entered into a new array, and points with different distances are represented with different colors. Objects closer than 1 m cannot be detected by LiDAR. This study represented various distances which are represented by different colors. The distance between 1 and 2 m is indicated in red, the distance between 2 and 3 m is indicated in yellow, the distance between 3 and 4 m is indicated in green, the distance between 4 and 5 m is indicated in light blue, and the distance that is greater than 5 m is in purple.

## 3. Experimental Results and Discussion

This experiment developed a modified Ackerman UMV to solve navigational and obstacle avoidance control problems. We designed several test situations to evaluate the navigational and obstacle avoidance ability of the Ackerman UMV. The experiment was divided into a navigation experiment and an obstacle avoidance experiment.

### 3.1. Navigation Experiment Results

**Figure 11:**
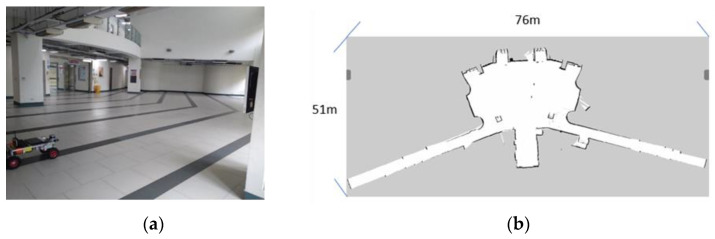
(**a**) Experimental verification scene. (**b**) Mapping of experimental scene using SLAM.

Figure 11 presents the experimental verification scene and the map constructed using SLAM. First, the researchers established two sets of starting points and destinations in the corners of the map. The navigation effect is more easily observed with a longer navigation distance; no obstacles were placed in the path. Speed and steering control (v, ω), distance traveled, and time were recorded. The trajectory of the moving distance is calculated by summing the distance between the real-time Ackerman UMV coordinates.

**Figure 12:**
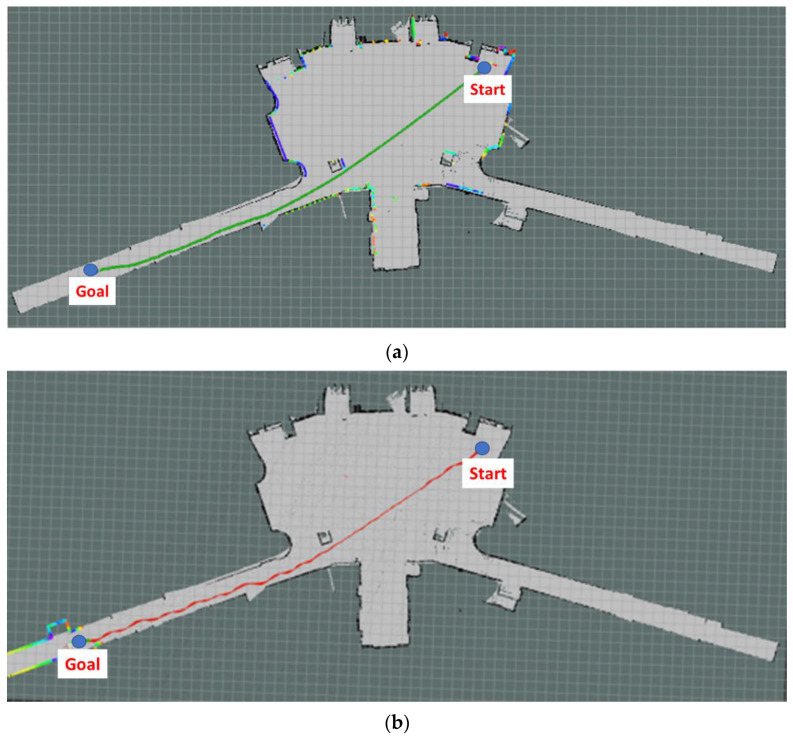
(**a**) Navigation path planned by the system. (**b**) Actual moving path of the Ackerman UMV.

The Ackerman UMV first located its own position on the map and was given the destination coordinates. According to the starting point and the goal point, the Ackerman UMV implemented the navigation algorithm and the cost map for navigation control and generated a predetermined path. Figure 12a is the navigation path planned by the system. After the Ackerman UMV generated the navigation path, the TEB algorithm was employed to plan the route of the Ackerman UMV based on this path and the real-time updated cost map. Figure 12b is the actual moving path of the Ackerman UMV. The experimental Ackerman UMV traveled a total distance of 49.83 m in 76 s.

**Figure 13:**
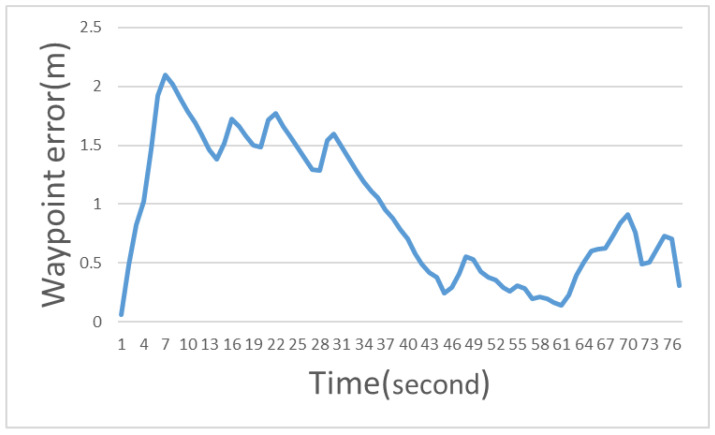
Waypoint error curve.

The waypoint error is the difference between the actual moving trajectory and the path planned by the system; it is used to evaluate the performance of the Ackerman UMV trajectory. The formula is expressed as follows:

(4) $$Waypoint\;error=\sqrt{{\left(nx-rx\right)}^{2}+{\left(ny-ry\right)}^{2}}$$

where *nx* and *ny* are the *x*-axis and *y*-axis coordinates of the path planned by the system, and *rx* and *ry* are the *x*-axis and *y*-axis coordinates of the actual moving track. The waypoint error curve is illustrated in Figure 13. The average error was 0.935 m.

**Figure 14:**
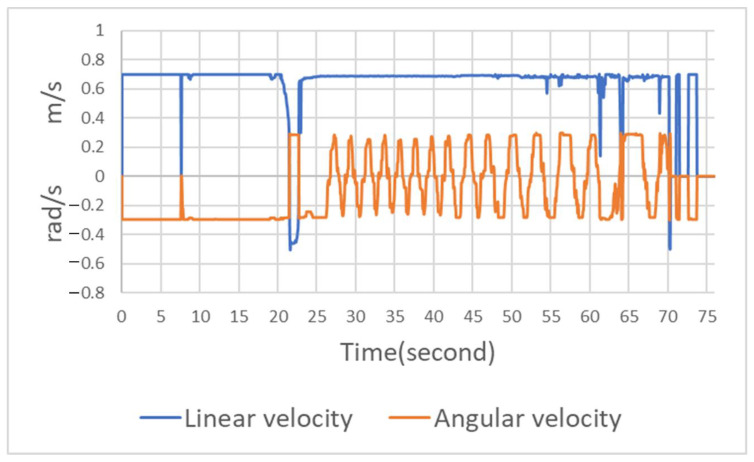
Speed and steering of Ackerman UMV.

The speed and steering of the Ackerman UMV are presented in Figure 14. The blue line indicates linear velocity where positive values are forward acceleration and negative values are backward acceleration; the orange line indicates angular velocity where positive values are deflections to the left and negative values are deflections to the right. This figure reveals that when the Ackerman UMV travels in a wide area, the speed and steering are relatively stable. When the Ackerman UMV travels in a narrow area, the speed and steering oscillate but do not affect the forward movement of the vehicle. The navigation algorithm has a recovery mechanism. When the algorithm detects that the Ackerman UMV cannot complete a task, it moves the Ackerman UMV backward, allowing the task to proceed. In this figure, the linear velocity during reverse indicates a negative value.

### 3.2. Obstacle Avoidance Experiment Results

This study implemented two obstacle avoidance experiments, the first with static obstacles and the second with dynamic obstacles. The starting point and purpose of the experiments were the same, and the speed and steering control (v, ω), moving distance, and time of the Ackerman UMV were recorded.

#### 3.2.1. Static Obstacles

**Figure 15:**
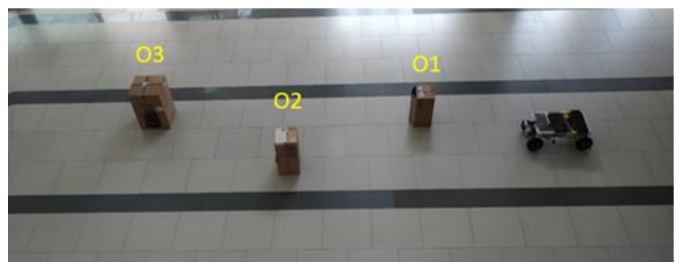
Top view of static obstacles in experimental field.

This study developed a short-distance assessment to evaluate the obstacle avoidance ability of the Ackerman UMV. Three obstacles were placed in the path of the Ackerman UMV on the way to its destination. Cardboard boxes placed crosswise were used as obstacles. The length, width, and height of the three cartons were 50 × 29 × 25 cm^3^ (O1), 58 × 30 × 30 cm^3^ (O2), and 63 × 43 × 43 cm^3^ (O3). Figure 15 presents a top view of the static obstacles in the experimental field.

**Figure 16:**
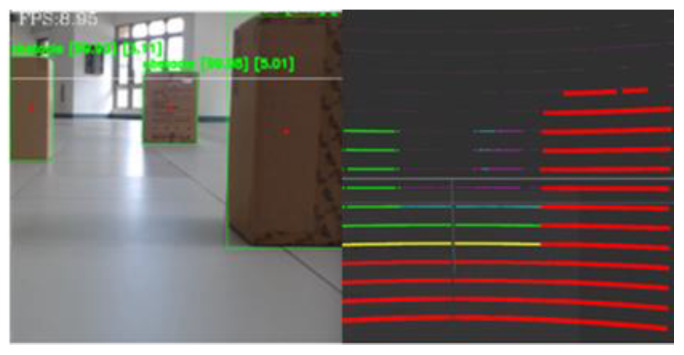
Combined LiDAR and imaging for static obstacle detection.

Figure 16 presents the combined LiDAR and imaging technology for static obstacle detection. This figure presents the category of the detected object, the confidence level of the category, and the point cloud information of the LiDAR. Therefore, the combination of LiDAR and imaging technology can be employed to identify objects and the distance of each object. Table 1 compares the actual distance and the distance obtained by the LiDAR between the three cartons and the Ackerman UMV. The average distance error using LiDAR was 0.03 m when the Ackerman UMV moved from start to destination. This result demonstrates that the distance obtained by LiDAR was quite accurate.

**Figure 17:**
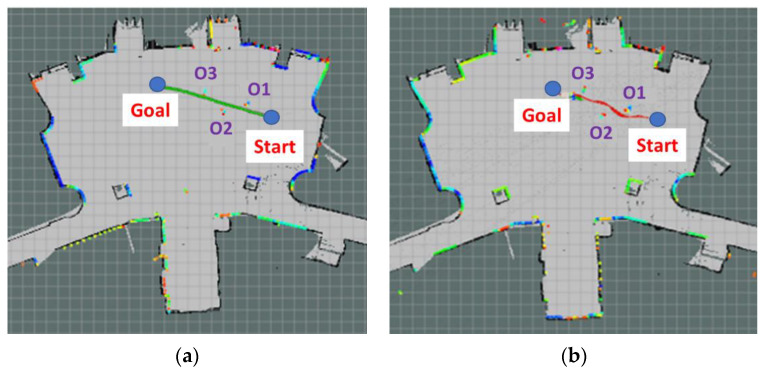
(**a**) Navigation planning path of the system. (**b**) Moving path of the Ackerman UMV with static obstacles.

Dijkstra’s algorithm determines the shortest path based on the cost map of the original map and between the starting point and the target point, but it does not consider real-time changes in the map. Figure 17a is the navigation planning path of the system. During the experimental navigation, the Ackerman UMV first generated a navigation path according to the obstacles, which was then optimized using the TEB algorithm. The colored dots on the map indicate reflections of the LiDAR sensor, which can detect the position of obstacles. Figure 17b illustrates the motion path of the Ackerman UMV with static obstacles. The Ackerman UMV traveled a distance of 10.7 m in 16 s.

**Figure 18:**
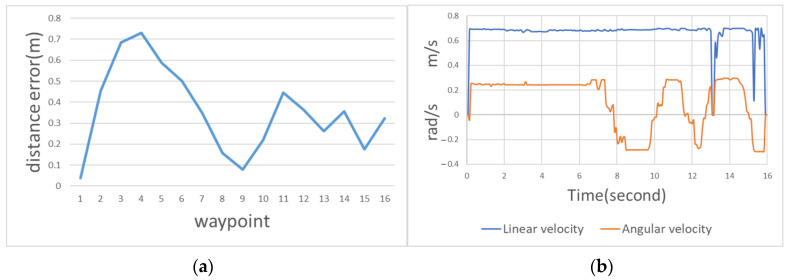
(**a**) Movement error curve with static obstacles. (**b**) Speed and steering of the Ackerman UMV with a static obstacle.

Figure 18a is the movement error curve with static obstacles. This figure indicates that the first turn was larger and had a higher error. The average error of the entire moving path was 0.357 m. Figure 18b illustrates the speed and steering of the Ackerman UMV with a static obstacle. This figure indicates that the route primarily passed through three large bends (i.e., bypassing obstacles) to reach the destination. The steering limit of the wheels and the expansion radius of obstacles required additional time for position adjustments.

#### 3.2.2. Dynamic Obstacles

**Figure 19:**
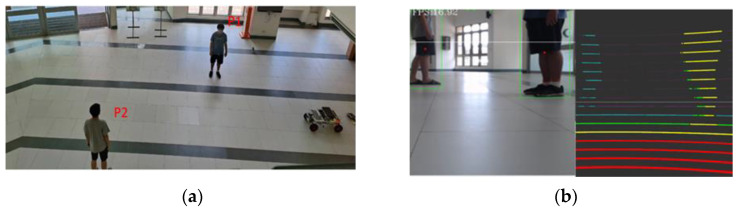
(**a**) Top view of dynamic obstacles in verification field. (**b**) Integration of LiDAR and imaging in dynamic obstacle detection.

The study then assessed the ability of the Ackerman UMV to avoid dynamic obstacles. An experiment was implemented in which two pedestrians passed in front of the moving vehicle. One pedestrian passed from left to right; the other passed from right to left. Figure 19a presents a top view of dynamic obstacles (i.e., the pedestrians) in the verification field. Figure 19b presents the integration of LiDAR and imaging in dynamic obstacle detection. Table 2 presents the error between the actual distance from the Ackerman UMV to the two pedestrians and the distance measured by the LiDAR. In this experiment, the Ackerman UMV needs to judge the moving directions of two pedestrians and formulate corresponding obstacle avoidance strategies. To detect and track moving objects effectively and quickly, the object detection method is adopted in this study.

**Figure 20:**
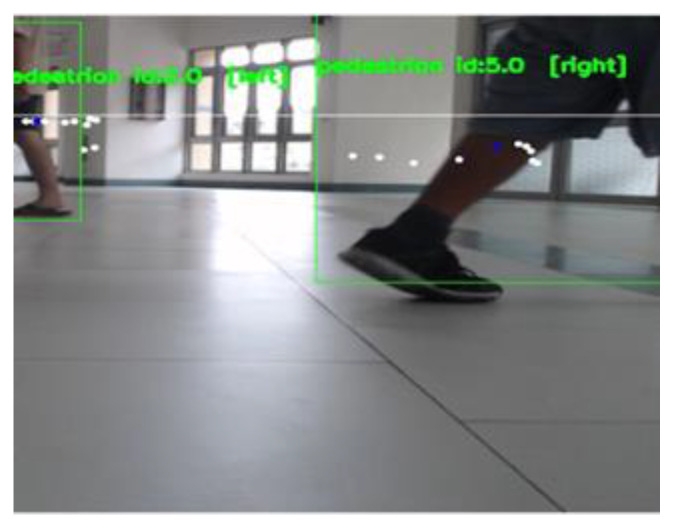
Tracking results of dynamic obstacles.

**Figure 21:**
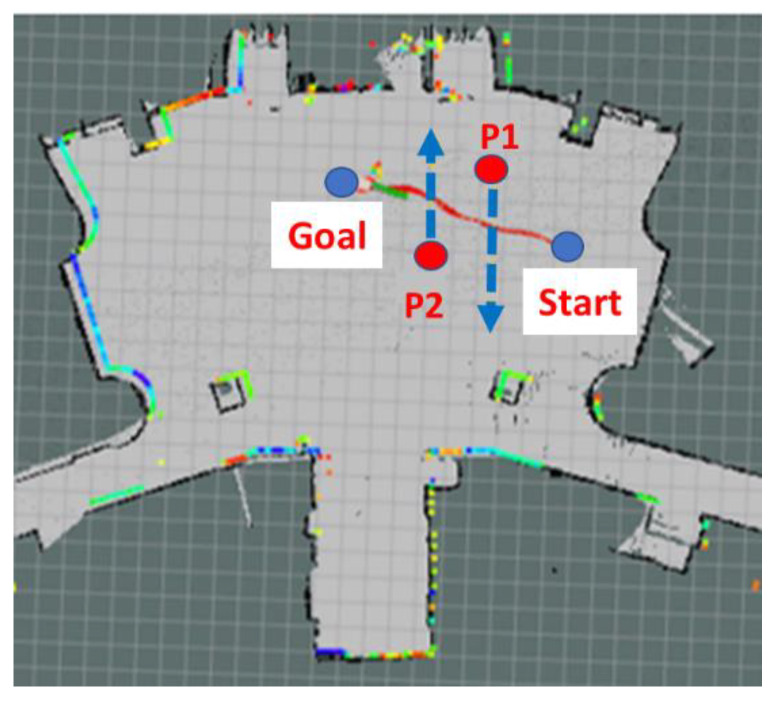
Path diagram for avoiding dynamic obstacles.

**Figure 22:**
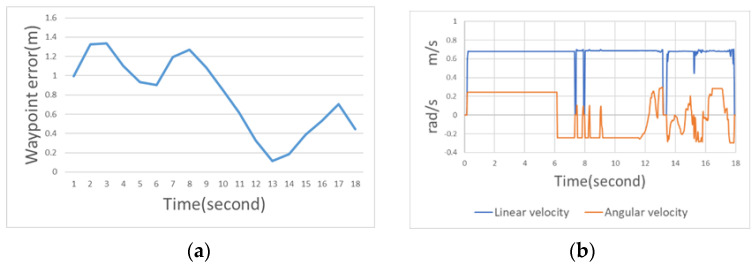
(**a**) Waypoint error curve of the Ackerman UMV. (**b**) Speed and steering of the Ackerman UMV when avoiding dynamic obstacles.

YOLOv4-tiny and SORT are used to track the movement trajectory of the center point of the dynamic obstacle (i.e., pedestrian) and judge the moving direction of the obstacle. The tracking results of dynamic obstacles are presented in Figure 20. Figure 21 is a path diagram for avoiding dynamic obstacles when the Ackerman UMV is in motion. In this figure, the direction of pedestrian movement is indicated by a blue line. During Ackerman UMV navigation, pedestrians will move according to the trajectory in Figure 21. The Ackerman UMV had a total travel distance of 8.07 m and a travel time of 18 s. When a pedestrian moves at a fast speed, the Ackerman UMV slows down, stops, and waits for the pedestrian to pass. However, when the pedestrian moves at a slow speed, the Ackerman UMV bypasses the dynamic obstacle. Figure 22a illustrates the waypoint error curve of the Ackerman UMV; the average error was 0.759 m. Figure 22b illustrates the speed and steering of the Ackerman UMV when avoiding dynamic obstacles. Experiments show that the proposed method can effectively avoid moving dynamic obstacles and can successfully reach the specified goal.

### 3.3. Discussion

**Figure 23:**
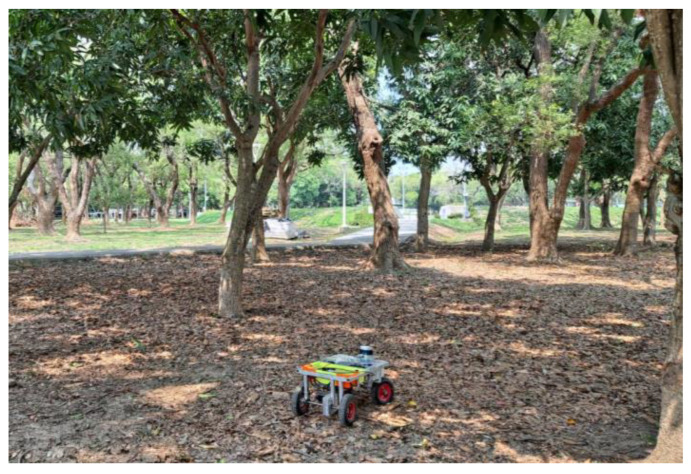
Experimental verification scenario.

In this subsection we will discuss the differences between single-sensor and multi-sensor fusion methods. Recently, multi-sensor fusion technology has been applied in unmanned vehicle systems. Caltagirone [32] developed an approach for road detection by fusing LiDAR point clouds and camera images. They point out that purely camera-based recognition underperforms severely. However, introducing LiDAR information can improve the overall recognition accuracy. Zhang [33] proposed a multimodal fusion method for the task of lane line segmentation and used the KITTI dataset to verify the performance. The experimental results show that the position of the lane line can be effectively segmented in the scene by using the multi-sensor information fusion method. Daniel [34] presents a solution for pedestrian detection which combines LiDAR point cloud data with multiple camera images. The results show that the proposed framework can accurately localize pedestrians in the range of 10 to 30 m, and the performance is evaluated by using accuracy, sensitivity, and precision. The above literature show that the fusion of LiDAR information and camera images can effectively improve object detection ability, thereby making unmanned vehicles more robust. Herein, we designed an experiment to compare the performance of multi-sensor fusion and single-sensor approaches. A forest is selected as the scenario for experimental verification, as shown in Figure 23.

A total of 500 point cloud data points and camera photos were collected, including trees and stone objects. This experiment compared the performance of single camera image, single LiDAR image, and LiDAR camera image fusion methods in object detection. The object detection results are shown in Table 3. As shown in the results in Table 3, the camera image is poor in detecting small objects. The reason is that the detection result is affected by the poor camera image due to insufficient light in the forest. In the detection results of LiDAR images, it can be observed that compared with camera images, the accuracy of small object recognition has improved a lot. However, the effect is not ideal in the detection of tree objects. By integrating LiDAR and camera images, it can be found that the performance has been greatly improved in both tree and stone object detection. It illustrates that the multi-sensor fusion method has better robustness than the single-sensor method. In terms of computing time, it takes about 55 ms for object detection on a single-sensor image. That is, the detected frame per second (FPS) is 20. Object detection with multiple sensors takes 71 ms. The multi-sensor fusion approach requires an additional 20 ms compared to the single-sensor approach. Overall, the proposed multi-sensor fusion approach is worthwhile to increase the accuracy from 50% to 80%.

## 4. Conclusions

This study proposed a fusion technique with heterogeneous imaging and LiDAR sensors in an Ackerman UMV. The major contributions of this study consist of the following: (1) The proposed system uses a camera to obtain real-time images, and YOLOv4-tiny and SORT are employed to classify, track, and detect the location of objects. (2) LiDAR is employed to obtain real-time distance information of detected objects which also combines camera images to improve detection accuracy. (3) The experimental results indicated an average distance error of 0.03 m using LiDAR and an average error of the entire motion path of 0.357 m. (4) The proposed Ackerman UMV successfully reaches its destination and instantly identifies static obstacles and pedestrians when in motion.

The proposed Ackerman UMV employs two heterogeneous sensors to detect obstacles. The control center collects a large amount of real-time data from these sensors, resulting in a long calculation time and subsequent system delay. Future research will implement an algorithm of the collected images and point cloud data using a field-programmable gate array to achieve real-time operation.

## Figures and Tables

**Table 1:** Comparison of the actual distance and the distance obtained by LiDAR.

Cartons (Length, Width and Height) (cm)	Actual Distance (m)	Distance Obtained by the LiDAR (m)	Error (m)
50 × 29 × 25	1.37	1.39	0.02
58 × 30 × 30	3.08	3.11	0.03
63 × 43 × 43	4.97	5.01	0.04

**Table 2:** Error between the actual distance and the distance measured by LiDAR.

Pedestrian	Actual Distance (m)	Distance Obtained by the LiDAR (m)	Error (m)
Pedestrian on the right	2.34	2.36	0.02
Pedestrian on the left	4.12	4.16	0.04

**Table 3:** Comparison results of different sensors in object detection.

Seneor	AP		mAP	Precision	Recall	F1-Score	Computing Time (ms)	FPS
	Tree	Stone						
Camera image	51.34%	11.11%	31.23%	55%	64%	59%	52.6	19
LiDAR image	30.93%	61.11%	46.01%	62%	35%	45%	55.5	18
LiDAR camera image	84%	63%	73.5%	84%	76%	80%	71.4	14

## Data Availability

Not applicable.
